# Case report: Two clusters of Creutzfeldt–Jakob disease cases within 1 year in West Michigan

**DOI:** 10.3389/fneur.2023.1134225

**Published:** 2023-03-20

**Authors:** Ling Ling Rong, Nicholas J. Lannen, Evan C. Tank, Jessica L. Feistel, Christopher J. Therasse, Anvita Potluri, Muhib Khan, Jiangyong Min

**Affiliations:** ^1^Department of Neurosciences, Corewell Health West, Michigan State University, Grand Rapids, MI, United States; ^2^Department of Radiology, Corewell Health West, Michigan State University, Grand Rapids, MI, United States

**Keywords:** Creutzfeldt-Jacob disease, cluster, rapidly progressive dementia, prion, real-time quaking-induced conversion, West Michigan

## Abstract

**Background:**

Creutzfeldt–Jakob disease (CJD) is a rare, rapidly progressive, and uniformly fatal neurodegenerative disease. The reported incidence of CJD is 1 to 2 per million people worldwide annually, with fewer than 1,000 cases in the United States per year. In this study, we report a unique case series on temporo-spatial clusters of CJD cases in West Michigan.

**Methods:**

A total of five CJD cases consisting of two temporal clusters were seen from July 2021 to June 2022 at Corewell Health West hospitals. All patients had brain MRI, EEG, and CSF tests. Four patients underwent autopsies.

**Results:**

All patients' MRIs showed characteristic CJD patterns. Four patients had positive CJD panels in CSF. One patient had typical CJD EEG findings. Four patients were confirmed as sporadic CJD by autopsy. All patients died within 3 months after CJD was suspected.

**Discussion:**

All patients lived within a 90-mile radius of Grand Rapids, MI, and two lived in the same county. West Michigan has a population of 1.6 million people, and the four counties where five patients lived have a combined population of 395,104, indicating CJD's new case rate of 3.1 and 12.5 per million people, respectively. Corewell Health is one of the three major healthcare systems in West Michigan. The actual incidence of CJD in West Michigan is likely even higher. This dense temporal and spatial cluster of CJD cases poses a serious public health challenge and warrants urgent investigation.

## Introduction

Creutzfeldt–Jakob disease (CJD) is a transmissible, rapidly progressive neurological disease, caused by misfolded prion protein in the brain (1). The reported prevalence of CJD is 1 to 2 per million people worldwide annually and less than 1,000 cases in the United States per year (2–5)^1^ CJD subtypes include sporadic, genetic, iatrogenic, and variant CJD with 85–90% of cases being sporadic (2, 6, 7). sCJD can further be divided into subtypes including MM/(MV)1, MM2, VV1, VV2, and MV2 based on disease-related prion protein features and prion protein genotype in the host at the methionine (M) and valine (V) polymorphic codon 129 (8–11)^1^. The clinical diagnostic criteria for probable sCJD are rapidly progressive dementia plus at least two of the following: myoclonus, visual or cerebellar signs, pyramidal/extrapyramidal signs, and akinetic mutism. Vertigo, headache, and neuropsychiatric symptoms can also present. Patients gradually lose mobility, speech, and progress into a comatose state (2, 6, 7, 12, 13). Despite extensive research since its initial description 100 years ago, CJD remains an incurable disease with a survival of 4–12 months from symptom onset in the vast majority of patients (2, 6, 7).

Over the past few decades, there have been increased reports on sCJD. Some studied regional or national geographical distribution or temporal occurrence, but their cases occurred during periods of 9 to 15 years (14–18). Few case series focused on cases with similar clinical presentation without patients' geographic information (19, 20), or on cases over 5 years in the same region (21).

Our case series includes two temporal clusters of CJD cases in one region. Within 1 year from July 2021 to June 2022, we observed five CJD cases at Corewell Health West Butterworth (BW) and Blodgett (BL) Hospitals in Grand Rapids, Michigan (MI). These two hospitals are 3 miles apart. All five cases had supportive brain magnetic resonance imaging (MRI), four of them had supportive cerebrospinal fluid (CSF) findings, and one case had a characteristic electroencephalogram (EEG) pattern. All patients died within 3 months after CJD was suspected. We report this dense temporo-spatial cluster of CJD cases to call for an urgent investigation by public health officials.

## Case series

*Patient 1:* A 67-year-old white woman who worked as a clinic manager was admitted on 14 July 2021 to BW due to rapid neurological decline. Her initial symptom was severe insomnia starting in mid-January 2021. By February 2021, her symptoms progressed to vertigo, diplopia, and imbalance. By May 2021, she was not able to function at work due to cognitive impairment. Her family noticed intermittent “childlike” behavior. On admission, she was fully alert, awake, and oriented with normal cranial nerves. Montreal Cognitive Assessment (MoCA) testing revealed profound deficits with a corrected score of 17/30.

*Patient 2:* A 78-year-old white man who was a semi-retired funeral home director was admitted on 31 July 2021 to BW for rapidly progressive cognitive decline along with dysfunctional gait, abnormal speech, and intermittent body jerking. In early May 2021, he started to have intermittent hand weakness, paresthesia, and forgetfulness. Due to unsteadiness, he began using a cane in June 2021 but quickly progressed to a walker. Outpatient electromyography and nerve conduction velocity (EMG/NCV) studies were unrevealing. MRI brain reported a 3-mm subacute infarct in left caudate head and ventriculomegaly. In the same month, he developed expressive aphasia progressing to a paucity of speech. Over 2 weeks, he had a catastrophic decline with excessive daytime somnolence. He was unable to perform activities of daily living and developed alternating urinary incontinence and retention. On admission, he was somnolent but easily startled by auditory stimuli. He followed limited, one-step commands and moved all extremities but was oriented to self only.

*Patient 3:* A 77-year-old white man who was a semi-retired attorney was transferred to BL for continuous video EEG monitoring on 03 May 2022 from an outside hospital (OSH), which was 75 miles east of Grand Rapids. In mid-March 2022, he complained of “brain fog” after he started medication for his newly diagnosed hypertension. The dyscognia persisted despite discontinuation of the anti-hypertensive. He developed visual disturbance and reported seeing his own fingers abnormally elongated, and his legs were fat and bowed. He was able to provide a full history and had an intact neurological examination on admission at the OSH 6 days before transfer to our facility. Brain MRI at OSH reported subtle cortical restricted diffusion involving both posterior temporoparietal and occipital regions. Levetiracetam was initiated after a 1-h EEG captured intermittent delta slowing over the left frontal region without rhythmicity. CSF at OSH was unremarkable except pending the CJD panel. Upon transfer to BL, he was awake and alert but with limited orientation, verbal output, and impaired abstraction. His motor and sensory examinations were intact.

*Patient 4:* A 78-year-old white woman and homemaker presented to the local emergency department on 03 June 2022 for cognitive decline over several months, accelerating over a few weeks before presentation. Her brain MRI reported multiple infarcts in different territories concerning global hypoxic ischemia; the on-call tele-stroke physician requested transfer to BW for further evaluation as the MRI pattern suggested CJD rather than hypoxic ischemia. The patient's initial symptom was intermittent forgetfulness, which started in August 2021 and had worsened since late December 2021. In March 2022, she developed “pressure in head” and complained “I do not feel my brain work.” The patient was diagnosed with anxiety and treated with anxiolytics without benefit. In late April 2022, gait abnormality and word-finding difficulties arose, and in May 2022, she developed auditory hallucinations. By June 2022, jerks in her upper extremities were noticed. Upon admission, she was oriented to person only and had impaired attention, reasoning, and verbal expression but had preserved motor strength and sensation.

**Figure 1:**
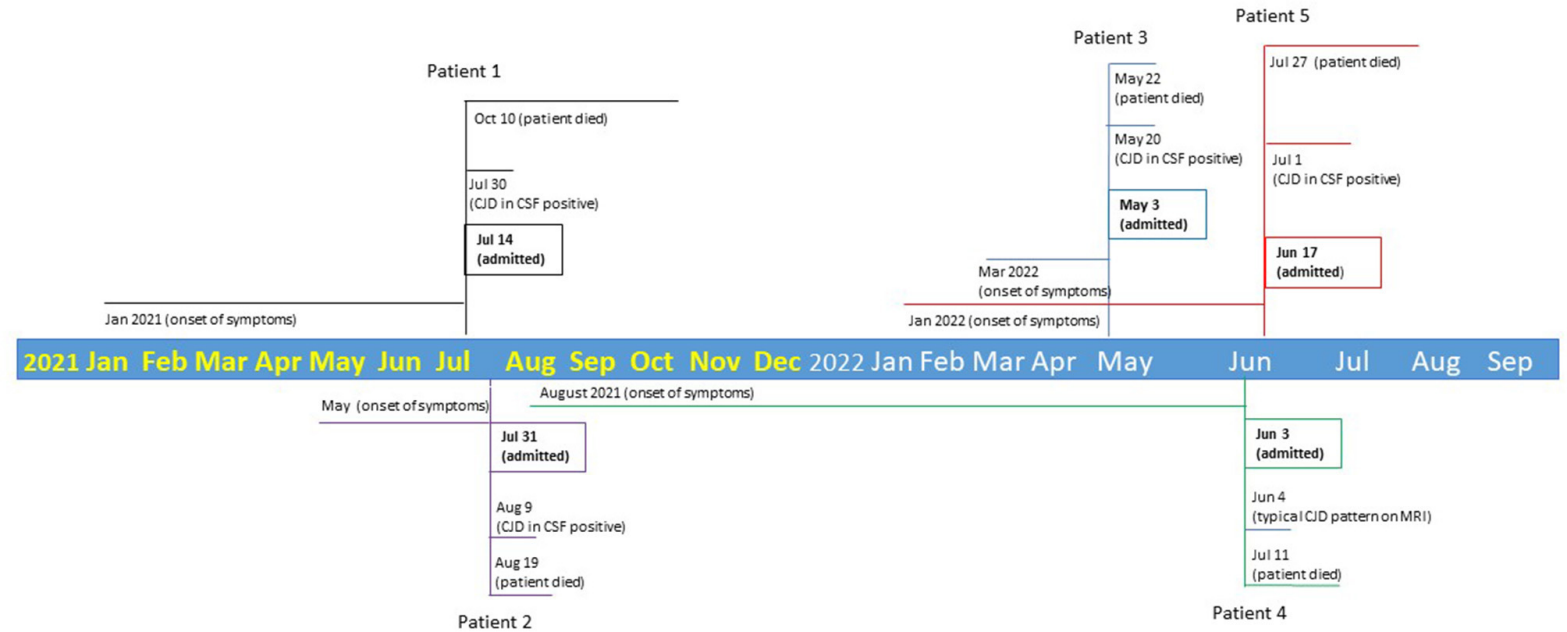
Timeline of two clusters of five CJD cases.

*Patient 5:* A 64-year-old white woman who worked as a nurse was admitted to BL on 17 June 2022 for rapidly progressive cognitive decline. Her initial symptoms were “brain fog,” dizziness, and fatigue, starting in January 2022. By late April 2022, she endorsed imbalance, diplopia, and multiple falls along with visual hallucinations, paranoia, and memory dysfunctions. On admission, she was awake, alert, oriented to self and could only recognize her close friends. Her cranial nerves were intact. She had mild proximal weakness in both bilateral upper and lower extremities. The vibratory sensation of bilateral lower extremities was impaired in a length-dependent distribution. She required stabilization on standing (Figure 1).

## Laboratory testing and results

**Figure 2:**
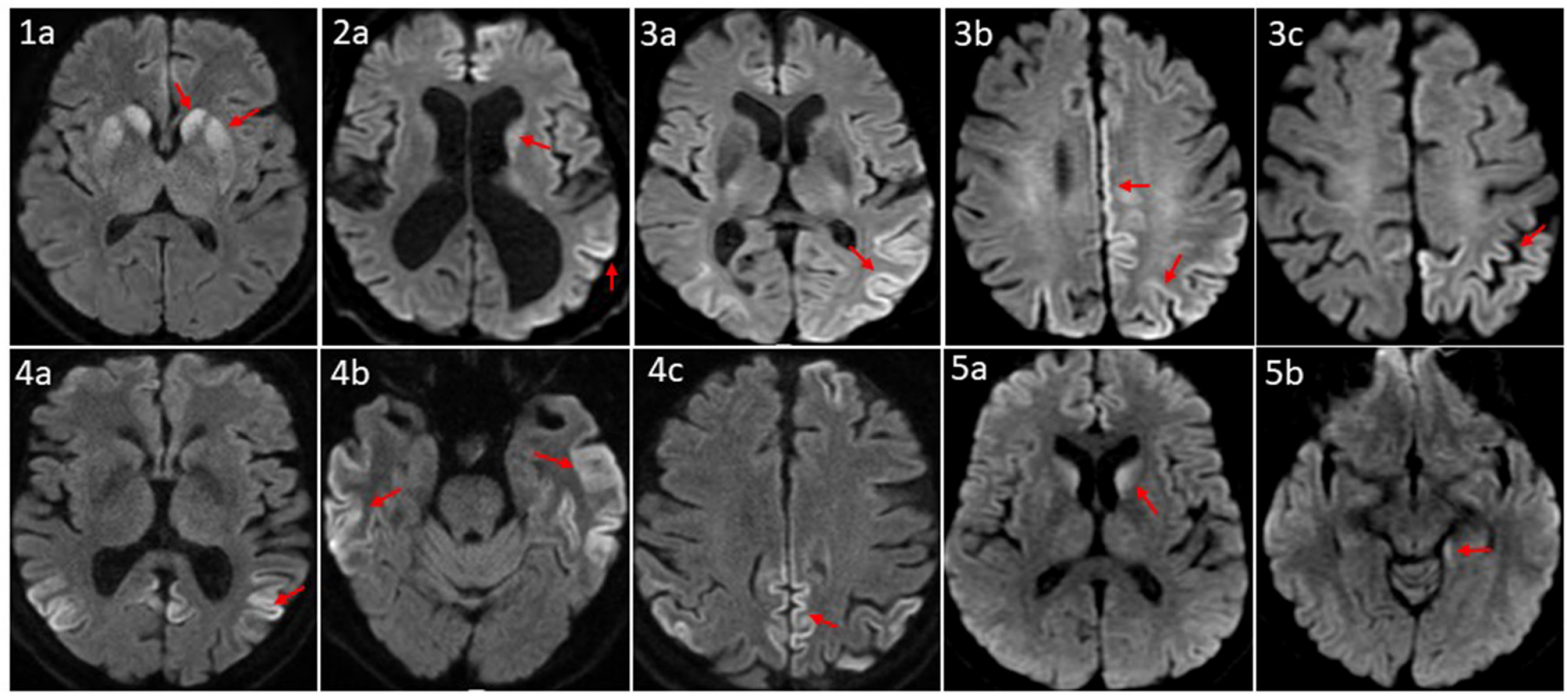
Axial MRI brain diffusion weighted images of five CID patients. **(1a)** Symmetric diffusion restriction in bilateral caudate nuclei and putamina. **(2a)** Subtle diffusion restriction involving the left caudate head, and left parletal cortex. **(3a–c)** Cortically based diffusion restriction involving the posterior left greater than right parietal lobes and paramedian left frontoparietal region. **(4a–c)** Intense cortically based restriction involving the paramedian and lateral parietal cortices, bilateral temporal cortices, and to a lesser extent the left greater that right frontal lobes. **(5a, b)** Relatively symmetric DWI within the caudate nuclei; subtle involvement of the posteromedial left hippocampus. Patient sequence was labeled from 1 to 5; red arrows point to sites of abnormal diffusion restriction.

All five patients had initial or repeated 1.5 or 3 Tesla brain MRI with and without contrast (w/wo) at BW or BL, respectively, revealing restricted diffusion and corresponding hyperintense T2 FLAIR signal involving bilateral caudate nuclei and putamina in a symmetric pattern (patient 1); asymmetric diffusion restriction signals in the cerebral cortex of cingulum, left temporoparietal lobes, and caudate nucleus (patient 2); prominent multifocal diffusion restriction involving the bi-hemispheric cerebral cortex, more posteriorly and on the left compared to the right (patient 3); symmetric cortical diffusion restriction involving paramedian, lateral parietal cortices, temporal cortices, and to a lesser extent in the frontal lobe with involvement of left greater than the right (patient 4); restricted diffusion in the bilateral caudate nuclei (left > right) and the left mesial temporal lobe, including the amygdala, the hippocampus, and the forniceal column (patient 5) (Figure 2).

**Figure 3:**
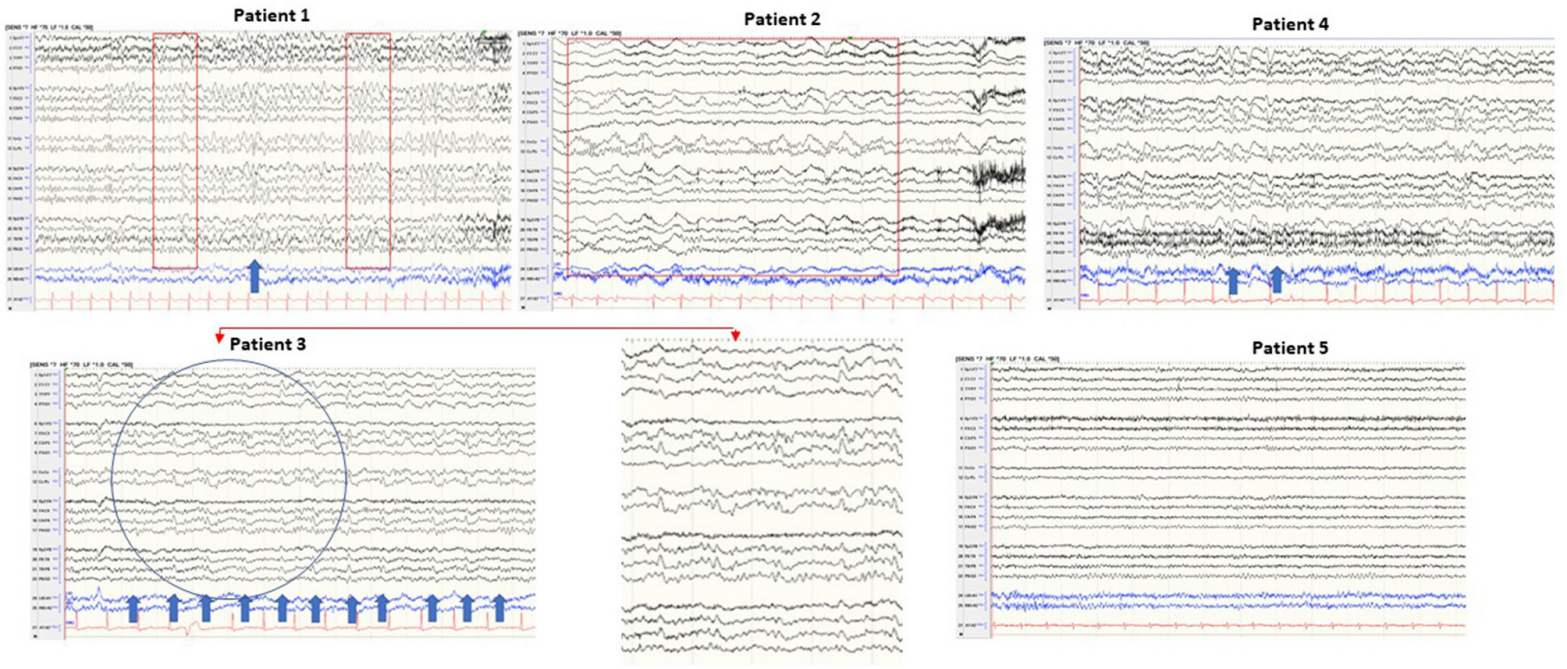
Typical samples of five patients in a standart anterior to posterior bipolar montage. Patient 1: background slowing and triphasic wave; patient 2: generalized rhythmic delta activity; patient 3: periodic sharp wave complexes (PSWC) at 1 Hz; patient 4: non-periodic triphasic wave; patient 5: normal EEG.

EEG was performed on all patients. The EEG of patient 3 showed periodic sharp wave complexes (PSWC), a characteristic CJD pattern. Other patients' EEGs were unremarkable (patient 5), had non-specific rare generalized periodic discharges with triphasic morphology (patients 1 and 4); moderate bitemporal slowing with a right predominance (patient 1); generalized rhythmic delta activity (patient 2) (Figure 3). Blood, CSF basic tests, and Mayo Clinic autoimmune encephalopathy panel in serum (ENS2) were negative. The autoimmune encephalopathy panel and paraneoplastic panel in CSF were all negative. All patients' CSF CJD panels from the National Prion Disease Pathology Surveillance Center (NPDPSC) reported >98% likelihood of prion disease except patient 4 whose CJD likelihood and 14-3-3 proteins were inconclusive, RT-QuIC was negative, and T-tau level was high (19937 pg/ml) (Table 1).

**Table 1:** Summary of patients' data, CSF CJD panel, and brain autopsy results.

**Case**	**Age**	**Gender**	**Profession**	**Admission**	**Expired**		**CSF**			**Autopsy**	**Final diagnosis**	**129 Polymorphism**
						**CJD probability**	**RT-QuIC**	**T-tau protein (pg/ml)**	**14-3-3 GAMMA (AU/ml)**			
1	67	Woman	Clinic Manager	Jul-2021	Oct-2021	>98%	Positive	>20,000	Positive	Yes	sCJD	VV2
2	78	Man	Funeral Home director	Jul-2021	Aug-2021	>98%	Positive	>20,000	Positive	No	NA	NA
3	77	Man	Semi-retired attorney	May-2022	May-2022	>98%	Positive	>20,000	72,061	Yes	sCJD	MM1
4	78	Woman	Homemaker	Jun-2022	Jul-2022	Inconclusive	Negative	19,937	Inconclusive	Yes	sCJD	MM1
5	64	Woman	Nurse	Jun-2022	Jul-2022	>98%	Positive	17,770	71,008	Yes	sCJD	VV2

## Treatment and final diagnosis

All patients (except patient 4) received empirical treatment: five doses of 1,000 mg methylprednisolone intravenously daily, followed by five rounds of plasma exchange (patient 1); one dose of 400 mg/kg intravenous immunoglobulin (IVIg) (patient 2); five doses of 400 mg/kg IVIg alone daily (patient 3); or with five doses of IV methylprednisolone daily (patient 5). Patients did not have any benefits from the aforementioned treatment. Patients died in October 2021, August 2021, May 2022, July 2022, and July 2022, respectively. Four patients underwent autopsy and genetic analysis (the family of patient 2 declined autopsy). No gene mutation was detected in any patient. Except for codon 129 polymorphism, no other polymorphism was found in any patient. The final diagnosis was as follows: patients 1 and 5 were sCJD-VV2, and patients 3 and 4 were sCJD-MM1 (Table 1).

## Discussion

All patients in this case series had a rapid cognitive decline. Two patients also had visual disturbances (particularly patient 3 who presented with impaired visual perception at an early stage), and the illness progressed rapidly, possibly representing the Heidenhain variant of CJD (12, 22). On admission, the patients were 67, 78, 77, 78, and 64 years of age, and the durations from the time of symptoms onset to death were 8, 4, 3, 11, and 6 months, respectively. This is mostly consistent with sporadic CJD, which has reported a range of 55–75 years of peak onset age and a median survival duration of 4–12 months (2, 5, 7, 23). The durations from the time when positive CJD in CSF was reported (in patients 1, 2, 3, and 5) or from the time when brain MRI showed typical CJD patterns (patient 4) to death were 72, 10, 2, 26, and 43 days, respectively. All were shorter than the reported typical 4 to 6 months from diagnosis to death (5, 23).

Brain MRI, EEG, and advanced CSF studies are the most utilized diagnostic tests for CJD (7, 24–26). Brain MRI with diffusion-weighted imaging (DWI) has a sensitivity of 67–91% (27, 28) and a specificity of 97% for diagnosing sCJD (27). With increased awareness of sCJD, its diagnostic criteria, and improvement in MRI accessibility and scan quality, the sensitivity of MRI for CJD could reach 99% (28). Our five patients' brain MRI revealed typical sCJD patterns, that is hyperintensities in the cortical gray matter (cortical ribboning sign) and the deep nuclei (basal ganglia and thalamus). The cortical ribboning sign was proposed to be the biomarker in the prodromal phase of sCJD diagnosis (29, 30). The MRI of patient 1 demonstrated a symmetric pattern of bilateral DWI and T2 FLAIR correlated signal in caudate nuclei and putamina. MRIs of patients 2, 4, and 5 revealed asymmetric, cortically based DWI changes in the cingulate, the caudate nuclei, and the left temporoparietal cortex, and the MRI of patient 3 showed multifocal cortically based DWI pattern, more on the left. Park et al. found that being greater than 60 years of age and diffusion restriction in the caudate nucleus and putamen were independent prognostic factors of shorter survival duration in patients with sCJD (27) with median overall survival of 1.7 months compared to 14.2 months in the intermediate risk group. Radiographically, our five patients belong to the high-risk group.

RT-QuIC is a breakthrough technology for diagnosing CJD with specificity reaching 99–100% (31–33). Its sensitivity can increase from 77 to 96% after modified techniques (31). Patients' (1, 2, 3, and 5) CSF CJD panel reported a likelihood of CJD of more than 98%, positive RT-QuIC, high T-tau protein, and positive 14-3-3 protein (more than 71,000 Au/ml in patients 3 and 5, and no titer reported in patient 1 and 2). The CSF of patient 4 was inconclusive for CJD likelihood and 14-3-3 proteins. Her RT-QuIC was negative, but T-tau protein was 19,937 pg/ml. As per NPDPSC test report, the sensitivity of RT-QuIC is lower when specimens are discolored by blood. Shir et al. reported that elevated CSF 14-3-3 and T-tau proteins as well as clinical symptoms such as myoclonus and visual or cerebellar abnormalities are associated with shorter disease duration (7), which held true for patient 3.

EEG has a lower diagnostic value when compared to brain MRI and CJD panel in CSF. The reported sensitivity of EEG-specific abnormalities to diagnose probable sCJD ranged from 38.2 to 68.75% (34, 35). However, the characteristic EEG finding in CJD, periodic sharp wave complexes (PSWCs), has 86% specificity (36) and 95% positive predictive value (37). Our case series confirmed low sensitivity and high specificity of EEG for diagnosing CJD. Of the five patients, four patients showed EEG abnormality (80%) with 20% specific (patient 3 showed characteristic periodic sharp wave complexes PSWC at 1 Hz, bi-hemispheric with left predominance) and 60% non-specific abnormalities (patients 1, 2, and 5). Mundlamurri et al. reported that in the early stage of sCJD, patients' EEGs can be normal or non-specifically abnormal (35). In very early phases (1.67 months after onset and before the emergence of generalized PSWC) of sCJD, the predominant findings of EEG can be (1) lateralized periodic discharges (LPDs), (2) central sagittal sporadic epileptiform discharges (CSSEDs), and (3) focal epileptiform discharges (38). It is suggested that the early presence of the PSWC pattern has a prognostic value because these patients have significantly lower average survival time (39). The EEG of patient 3 captured PSWC on day 48 after the illness onset. He died 19 days after the EEG was done and 2 days after positive CJD in CSF was reported.

Brain biopsy or autopsy remains the gold standard for final diagnosis. Four patients underwent autopsy and genetic analysis. All four patients had sporadic CJD, of the two most common subtypes, sCJD-VV2 in Patients 1 and 5, and sCJD-MM1 in patients 3 and 4. Different subtypes have different clinical and neuropathological features, as well as survival times and test results (8, 40, 41). The sensitivity of RT-QuIC for detecting MM1 and VV2 is high (96.3%) but can be negative for MM2 and VV1 subtypes (33, 42). Younes et al. reported that MM1, MV1, and VV2 are related to short duration/fast progression, while MV2, VV1, and MM2 are associated with long duration/slow progression (43). Our case series revealed that patients 3 and 4 were sCJD-MM1 but with an 8-month difference in survival length; patients 1 and 5 were diagnosed with sCJD-VV2 with similar survival durations, 8 vs. 6 months. Patient 2 had a short survival duration; unfortunately, his family declined an autopsy.

Geographical clusters of sCJD have been reported, but most clusters contained cases distributed over many years (14, 17, 18, 44–48), and few were temporo-spatial clusters. A French cluster of three cases of CJD occurring in 1998 reported that two of the patients lived in the same village. Molecular and phenotypic analyses showed both patients were homozygous for methionine at the polymorphic codon 129 but one patient was MM1 while another had mixed features of MM1 and MM2 both clinically and histo-pathologically (48). RT-QuIC was not yet invented. A Japanese cluster of three CJD cases occurred between 1988 and 1989 near Fukuoka city; no hospitalization time was mentioned in the report, nor were CSF studies or codon 129 polymorphism analyses done on these patients (49). A cluster of four cases in Burlington, Ontario, Canada, between April 1989 and April 1990 with two additional cases on further inquiry, and a cluster of seven cases in Nassau County, New York, between mid-June 1999 and mid-June 2000 (50, 51) were reported without genetic studies. Some clustering was found later to be an aggregation of genetic CJD cases (52, 53).

Our five cases in two clusters were seen within 1 year in Grand Rapids, Michigan. Cluster one included patients 1 and 2, seen within 1 month from July to August of 2021; cluster two included patients 3, 4, and 5, observed within 1 month between May and June of 2022. All patients lived within a 90-mile radius of Grand Rapids. No interpersonal connections were identified among them. All patients were white with differing professions (Table 1). None of them had a family history of Creutzfeldt–Jakob disease, or personal history of corneal transplants, craniotomy, administration of human growth hormone derived from pools of pituitary glands, or surgical procedure at the same facility. However, families of patients 1, 2, and 4 reported consuming venison. More intriguingly, families and relatives of these three patients reported additional (at least four) possible or probable CJD cases occurring between 2007 and 2022 in their friends or communities (unpublished data). One of the patients was a 63-year-old white woman and mayor, who lived 35 miles from patient 2, and died of CJD in March 2022. Thus, such a wave of dense temporo-spatial clustering of CJD in West Michigan is very unusual and alarming.

Our case series does not support that CJD incidence has no geographical differences (4, 54). West Michigan has 1.6 million people, and the combined population of four counties where five patients lived is 395,104 in 2022, which makes the CJD new case rate 3.1 and 12.5 per million people in West Michigan and combined four counties, respectively, which is higher than reported 1 to 2 per million people worldwide and 350–710 cases in the United States annually (2–5)^1^. Adding the cases reported by our three patients' families, the new case occurrence would be even higher. Michigan disease surveillance system (MDSS) reported 19 CJD cases by 31 December 2022 and only 12 cases in 2018, and this reflects a 58% increase^2^ We do not have enough evidence to conclude that our two clusters are purely due to heightened awareness, more sensitive tests, and better ascertainment, nor could we be certain that they just simultaneously occurred (55). Our study has several limitations, including an observational study, a limited time period, not using the conventionally used solar year period, and a relatively small population and area in West Michigan. As such, this case series highlights only a possible trend. More research and evidence are certainly required to reach a conclusion. We have planned additional retrospective studies, which we expect will surmount these shortcomings. Epidemiological surveillance, research, development of new diagnostic technologies, and public health endeavors are critical (4, 56).

## Conclusion

For five sCJD cases in two dense clusters within 1 year in Grand Rapids, MI is more than expected. Extensive screening in West Michigan may eventually arrive at a reliable incidence rate of CJD in this region. These two clusters along with additional cases reported by our patients' families warrant urgent investigation. Further research including epidemiological study regarding possible transmission events, common environmental factors that trigger CJD occurrence as well as continuous surveillance, and further improving diagnostic techniques are critical and necessary.

## Data availability statement

The raw data supporting the conclusions of this article will be made available by the authors, without undue reservation.

## Ethics statement

Ethical review and approval was not required for the study on human participants in accordance with the local legislation and institutional requirements. The patients/participants provided their written informed consent to participate in this study. Written informed consent was obtained from the individual(s) for the publication of any potentially identifiable images or data included in this article.

## Author contributions

LR: writing the original draft, selecting MRI images, and finalizing the manuscript. NL, JF, AP, MK, and JM: reviewing and editing. ET: selecting EEG pictures, draft reviewing, and editing. CT: selecting MRI images. All authors contributed to the article and approved the submitted version.

## Footnotes

^1^ https://case.edu/medicine/pathology/divisions/national-prion-disease-pathology-surveillance-center/human-prion-diseases

^2^ https://www.michigan.gov/mdhhs/-/media/Project/Websites/mdhhs/CDINFO/WSR/Current_WSR.pdf?rev=2219a79dac8f486fa870c4afcb339848&hash=F23C8EB5D0CCD5378F6A5AE8CF1CB954
